# Food Safety Gaps between Consumers’ Expectations and Perceptions: Development and Verification of a Gap-Assessment Tool

**DOI:** 10.3390/ijerph17176328

**Published:** 2020-08-31

**Authors:** Paohui Lin, Hsientang Tsai, Tzuya Ho

**Affiliations:** 1 Department of Business Management, National Sun Yat-sen University No. 70, Lien-Hai Rd., Kaohsiung 804, Taiwan; b827@ms35.hinet.net (P.L.); htt@mail.nsysu.edu.tw (H.T.); 2 Business School, Shantou University, 243 Daxue Rd., Shantou 515063, Guangdong, China

**Keywords:** food safety, food safety gap, importance–performance analysis, consumer perceptions, consumers expectations

## Abstract

In recent decades, food safety has become a major concern due to frequent food safety incidents in many countries. This may lead to increased health risks associated with low quality food consumption, thereby reducing consumer trust in food safety. A better understanding of consumer perceptions of food safety can improve indicators that do not meet consumer expectations. We propose a food safety gap model with four gap-construct based on consumer expectations and perceptions. The model was empirically tested through a survey of 25 items, and then assessed for gaps through the importance–performance analysis (IPA). From a sample of 697 Taiwanese consumers, we found a huge gap between consumer expectations and perceptions related to food safety. More importantly, the results of the IPA indicate that most items must be immediately improved, which is vital in order to mitigate the risk of food safety.

## 1. Introduction

Food safety has become an important public health concern, so it is controlled and regulated by the government. Governments have taken measures to manage issues, and many previous studies have also explored these mechanisms, including governmental policies and regulations on organic farming [1], the impact of foodborne poisoning caused by food contamination [2], a novel coronavirus (COVID-19) caused by microbial contaminants in game meat [3], concerning the risk of animal diseases such as avian flu, swine flu, mad cow virus [4,5], safety assessment of meat products [6], and food inspection standards, such as instrumental detection methods based on the nuclear magnet resonance [7] or low field nuclear magnetic resonance [8] to assess the adulteration of oils and fats. It is hoped that these approaches can be used to ensure a baseline level of food safety.

In academia, various researchers have studied complex food safety issues, each with its own terminology and classification, for example, Wu and Hsiao [9] used a failure mode and effect analysis (FMEA) method to evaluate risk in food cold chains for chilled or frozen food companies in Taiwan; Lai et al. [10] estimated on disease burden from foodborne illnesses in Taiwan from 2012 to 2015; Wu, Lin, and Chen [11] investigated food safety risks in China; Whitworth, Druckman, and Woodward [12] proposed the categorization of food scares; Jespersen, MacLaurin, and Vlerick [13] developed the food safety desirability response scale; Park, Kim, and Bahk [14] analyzed South Korean food safety incidents between 1998 and 2016; Nayak and Waterson [15] studied the food safety culture of a group of industry stakeholders in the UK; Wongprawmas and Canavari [16] provided food safety label categories that are indicators of Thailand consumers’ willingness to pay. Despite the increased academic interest in food safety issues in recent years, food safety incidents remain problematic and have not declined significantly over the past decade.

Food safety incidents cause economic losses, social disruption, and also have environmental impact, so manufacturers involved in the food supply chain and associated governmental departments are striving to reduce both the frequency and severity of their occurrence. However, many of these incidents can be traced back to food manufacturers and their non-compliance with food hygiene and/or food safety procedures. For example, Sun et al. [17] pointed out that street food vending in the night markets presents public health challenges, with evidence of diarrheal diseases. Park et al. [14] found that between 1998 and 2016, South Korea had an average of 4.3 food safety incidents per month, which had both local and international impacts.

According to Peng et al. [18], Taiwan has also faced food safety incidents in recent years, with adulteration involving illegal additives being the most common: black tea with coumarin, emulsifiers with plasticizer, edible oils with Cu-chlorophyll, lard with recycled cooking oil, and antibiotics (tetracycline) in honey. It is worth noting that the Taiwan Food and Drug Administration (TFDA) has established official regulations and analytical methods to assess food quality and determine whether food is adulterated. However, many food safety incidents still occur, although the cause of each event may vary by product, time, and geographic distribution, but a pattern can be seen between certain events. Therefore, it is useful to classify consumers’ similar perception gaps when developing strategies to reduce food safety scares.

Parasuraman, Zeithaml, and Berry [19] noted that the consumer expectations will be influenced by past experiences, personal needs, and word-of-mouth. Indeed, there are various attributes of safety food. With the improvement of food safety awareness, more consumers are willing to pay higher prices to buy safe foods. For example, products with food traceability, food certification mark, and labeling of product ingredients. Parasuraman, Zeithaml, and Berry [19] studied consumer perception models (called PZB model), which is the most cited literature on service quality gaps. The original PZB model proposed five gaps to describe the service quality model by conducting in-depth interviews with fourteen executives from four service companies and service users. The PZB model is very attractive because it can reasonably explain the gap between perception and expectation. However, the model concerned the perceived discrepancy between management and customer. The gap is strongly managers-oriented and not particularly applicable in the context of food safety. Thus, we propose a food safety gap model that uses the concept of the PZB to describe the food safety gap between consumer perceptions and expectations, and develops a multiple-item instrument to measure these gaps.

## 2. Conceptualizing Food Safety Gap

Prior research focused on consumer perceptions of food safety risks [11], food-related hazards [20], trust of food [21,22], and consumer acceptance of food safety incidents [23,24]. Recent studies have developed several instruments to measure perceptions of food safety in food-related organizations, such as food safety climate [25], and food safety culture [26].

**Figure 1:**
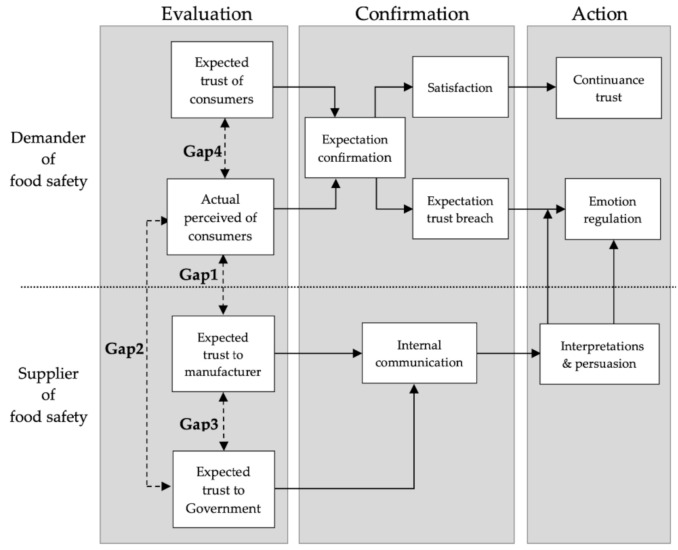
Food safety gap in the Evaluation-Confirmation-Action (ECA) model: A conceptual model that defined the consumer’s food safety gap and psychological activity.

Nonetheless, based on the consumer-oriented context, the concepts of food safety gaps are still ambiguous and no unanimous definition has been found. Drawing on food safety related literature and expert views, we have developed a conceptual model of psychological activity of food consumers to explicate the key determinants of food safety gaps, and define the main concepts which are present in this study. The psychological activity of food safety perceptions includes three stages: evaluation, confirmation, and action. We called this the ECA model and depicted this conceptually in Figure 1.

The evaluation stage is subdivided into four gaps between consumers’ expectations and perceptions. This process is derived from the gap concept of the PZB model [19]. Confirmation and action stages can be divided into the expectation of confirmation or not as the antecedent of subsequent perceptions. The upper part of the framework refers to related concepts of the expectation confirmation theory (ECT; [27,28]). This explains how consumers evaluate their food safety experience and how this evaluation affects their decision to continue purchasing (e.g., expectation confirmation → satisfaction → continuance trust).

The lower part depicts the psychological contract breach [29]. Reneging and incongruence are two root causes of perceived psychological contract breach. For example, an obligation exists but knowingly fails to meet that obligation, or made an explicit promise and then failed to uphold that promise. However, the perception of contract breach will not necessarily lead to intense emotional reactions. Whether this cognitive perception of breach will lead to an emotional response, and the intensity of this reaction, depends on the interpretation and persuasion process of the defaulter, whereby the consumers attaches meaning to the perceived breach (e.g., expectation disconfirmation → expectation trust breach → emotion regulation ← interpretation/persuasion).

The definition of the ECA model in our study includes the gap of expected and perceived, which then affects cognition attitude and subsequent behavior intention. After experiencing the first-stage of the evaluation and finding a perceived gap, if consumers’ perceptions of food safety are much lower than expected, consumers are exposed to food safety gaps and vulnerabilities. That is, the importance of food safety and gaps are the main reasons for this study. Study the expectations versus the actual perceived disparities at the consumer’s level and unravelling human perceptions in the context of food safety is important as both might affect the validity of the evaluation stage. This study emphasizes a consumers’ perspective on food safety being subjective and experiential. This approach aims to explore gaps on how consumers actually perceive food safety in terms of an evaluation of its expected trust. Therefore, we will not only give a brief overview of prior research on food safety but also discuss the food safety gap model, and assessment tool.

### Food Safety Gap (FSG): Components and Definition

Food safety is defined as a necessary condition to guarantee the health of consumers [30], which is considered as the basic rights of consumers. Food safety specifications and laws are among the instruments used from manufacturers and governments to discuss safety issues in the food supply chain [31]. Governments often set food-related regulations or standards to figure out the baseline level in food safety, which can protect consumers from food safety issues [32], this includes a number of routines that should be followed to avoid potential health hazards.

Nelson [33] argued the food has credence-attributes in the consumption market and may face the asymmetry of product information because consumers cannot see or verify, either before or after purchase. If this is not a priority, this can cause serious damage in food safety. However, giving credence-attributes can be costly, and firms can also incur fixed costs associated with providing accurate information [34], so reducing credence-attributes can be one of the means to improve performance.

The tracks within this line of thought are safety between the government and industry, and then between industry and the market, and then between the market and the consumer. Specifically, a lack of food safety can be the main reason for the gaps in perceptions that consumers have. Therefore, Gap 1 to Gap 4 illustrates the difference between consumers’ actual perceptions and expectations concerning food safety in the first-stage of the ECA model. The gap model further constitutes a theoretical explanation for the mentality of food safety, which is the main focus of this study.

Among these four gaps of food safety trust, ‘GAP’ stands for the expected difference between consumers, manufacturers, and government. Gap 1 shows the difference between consumers’ actual perceived and expected trust towards manufacturers. Before selling food, a company’s management essential to understand the characteristic of food safety to fill consumers’ needs, due to the affected consumers’ evaluation of food safety.

Gap 2 describes the consistency of trust between consumers and the government in food safety, which in turn forms food safety regulations to protect consumers. It is because the commitment and policies communications by government can affect consumers’ perceptions regarding food safety. Gap 3 is the discrepancy between food manufacturers and what is promised via external regulations put in place by the government. Various situations, such as legal conditions and regulatory constraints, may lead to differences between expected trust from manufacturers and governments by consumers and what they perceive these established regulations for food safety.

Gap 4 is the difference between consumers’ perceptions and expectations of food safety. The gap is concept-oriented, especially for food safety. Gap 4 may also be affected by gaps 1, 2, and 3. The means that the direction and size of each gap will have an influence on food safety. The reasons for the gap include (a) unrealistic food information, (b) public anxiety over food safety incidents, and (c) poor enforcement of food safety regulations. Hence, further research is needed to help consumers understand their gaps of food safety.

## 3. Developing a Self-Assessment Scale for Food Safety Gap of Consumer

In order to develop the Food Safety Gap Scale (FSGS) to measure the gap between consumer expectations and food safety-related concepts, we collected 40 items related to the basic attributes of food quality from previous studies. We divided these 40 measurement items into four dimensions, which include assurance, commitment, regulation, and assessment. The explanation of each dimension is as follows.

### 3.1. Assurance: The Expectation Gap of Consumers and Manufacturers

Assurance—This is related to measuring the objectivity or clear evidence of food, such as food label information, appearances being kept intact, food certification marks, labeling of product ingredients, and food traceability [35]. These items which were classified as “food manufacturers have the control, honest, and sufficiently open about the safety of food” (A1-3) are based on the consumers for confidence-in-food manufacturers questionnaire [36].

For consumers, manufacturers must prove that food safety and hygiene are more important than productivity and cost savings. Although this notion already is ingrained in the consumers’ perception, food safety goals often are in conflict with the financial performance of a manufacturer [37]. Therefore, if the manufacturers’ ethical beliefs and values of food safety align with consumers, they will be more motivated and exceed expectations.

This will then decrease negative perceptions of food safety for consumers such as item A9 “Absence of a code of ethics in enterprises makes you suspect the safety of the food with additives.” [38], and item A10 “alert and attentive to potential problems and risks related to hygiene and food safety” [39]. In addition, according to Taylor’s survey items, where framed in the context of food safety: “the food product is labelled with all necessary information” (A4) as well as “ The food producer (A5)/shop or retailer (A6) maintains control over the hygiene” [40], these items were adopted.

Another indicator that manufacturers face with food safety issues is the assurance of trust. As argued by Morgan and Hunt [41], trust and confidence plays an important role in the business/consumer relationship, yet this trust appears only when the supplier satisfies the needs of the consumer [42]. This sense can bring about increased motivation on the part of the consumer, and it also means that people “trust Taiwanese food manufacturers to produce safe foods” (A7) and “trust processors to honestly convey the country of origin and the product’s ingredients” (A8) [21].

As described above, items of the food safety gap between consumers and manufacturers are integrated into the first version of the self-assessment scale in Table 1.

### 3.2. Commitment: The Expectation Gap between Consumers and the Government

The commitment to food safety through government is a measure which consumers consider regarding their own values and beliefs about food safety, and if they are aligned with those of their own government. Previous research has identified the relationships between the positive and negative effects of organizational commitment across a wide range of different contexts [43,44]. Nevertheless, the extent of the changes brought upon by the commitment of government is yet to be explored.

In this case, the component commitment of this study is to identify the impact of government commitment on consumers, and to assess the expectation gap between consumers and government. Based on the leader–member exchange (LMX), the commitment in an environment of food safety is affected by the government–consumer exchange (GCX). This notion is inferred from the quality of the social exchanges between government and consumers. Regarding GCX, a high GCX can result in better engagement in commitment concerning regulation and policies.

The term “commitment” is used to describe food safety, the purpose is to reduce the social panic that may be caused by contaminated food, and how to effectively promote policies to attract consumers [45]. For example, item C1 “Government’s regulation on additives is a lack of effectiveness” [38], item C2 “government set clear objectives”, and item C3 “government strive for continuous improvement” [25].

Every time the government participates in critical food issues, it must prove the significance of their regulations and how they impact food safety. Therefore, we introduced item C4 “government clearly considers food safety and hygiene to be of great importance” and item C5 “government acts quickly to correct problems/issues” [46]. We argued that a high GCX also means that communication from government to consumers is a major factor.

Generally, consumers expect that food will not harm their health or the environment. Consumers’ trust in regulators is vital in building and maintaining confidence in food safety [47]. Therefore, Griffith et al. [39] conceptualized that consumers’ confidence in food safety is influenced by their trust in government, food safety risks and food risk controls, such as items C6, C7, and C8.

When consumers cannot maintain the control of food quality they are consuming, the trustworthy relationship with government is considered a good deputy for controllability [48]. The choices associated with the assessments of food risks result in a very time-consuming process. So, people need to delegate control with certain situations and let other trusted characters act on their behalf [49].

In this sense, the level of food safety control handing as a determinant of food quality is based on government regulations, specifically, the safety controls required by law for public hygiene, risks related to manufacturers’ food production, and certifying bodies’ indirect controllability. For these reasons, it is important to know how much trust the public has in the government’s food regulations to cope with food risks, such as development items C9 [50], C10 [51], C11 and C12 [52] (see Table 2).

### 3.3. Regulation: The Expectation Gap between Government and Manufacturers

The government should design regulations and standards related to hygiene and food safety, and ensure that manufacturers know their roles and responsibilities. At the regulatory level, Taiwanese laws abide with the strict regulatory framework established by the United States and the European Union (e.g., Hazard Analysis and Critical Control Points (HACCP), Genetically Modified Organisms (GMOs), and Regulation (EC) No. 178/2002) to trace food safety. For this reason, item R1 “Taiwanese authorities enforce strict hygienic standards for food” [40] was retained.

In addition, the nature of trust is very complex [53], such as the recognized qualities of Taiwanese local gastronomic products, but Taiwanese may also have doubts regarding the safety of food. Specifically, over the various food safety incidents which may have led to lower consumer confidence and an increase in food scares. On the one hand, food scares can be interpreted with more and more people having anxiety over food safety incidents, and this anxiety is closely correlated with media attention given to such events [54]. On the other hand, media information about the relationship between food safety and consumers is often highly distrustful compared to other sources, but it is also a primary source of information for many consumers [49].

In general, if government authorities appear willing to disseminate relevant regulatory information regarding food safety, consumers will be have more “trust in imported food, and feel it is safe” (R2), “trust the food inspection schemes adopted” (R3), and “trust the food safety standards adopted” (R4) [21]. If “governments communicate regularly with the public (R5) and food manufacturers (R6) about hygiene and food safety” [50], this process creates a short-term positive impact upon consumer consumption behavior.

Additionally, whether these violations of public health will be corrected immediately have also caused significant consumers concern. Therefore, Guchait, Neal, and Simons [37] proposed a causal sequence illustrating the importance of serious (R7) and substantial (R8) food safety violations. Each of the above these items are included in Table 3.

### 3.4. Assessment: The Gap between Expectations and Actual Perceptions of Consumers

Expectation-disconfirmation theory (EDT) [27] has been utilized by researchers to understand consumer satisfaction, intention to purchase again in the future, and complaining behaviors, and is an extension of cognitive dissonance theory (CDT) [55]. CDT explains how discrepancies between one’s cognition and reality change the individual’s later cognition and behavior. In the context of food safety, cognition refers to one’s food risk opinions [56], food consumption values [57], affective attitudes of food [58], and food safety knowledge [59], while behavior refers to actions started in response to this cognition or individual’s evaluation of such behavior [55].

Food safety is a social risk associated with human factors, therefore, the relationship between expectations and actual perception is complex. Using EDT to depict a psychological process of individual behavior whereby consumers generate initial expectations about a food product, experience its usage over time, and then form actual perceptions of the food product itself. Although earlier research explained the gap between food safety attitudes and behavior [60,61], it did not measure the consumers’ observed expectations of change in terms of cognitive dissonance.

The gap between consumers’ original expectations and actual perceived existed in the disconfirmation construct. Cognitive dissonance may be positive or negative depending on whether the actual perception is above or below initial expectations, and is viewed as a deviation of initial expectations. Indeed, some contend that the gap is ambiguous, and the effect appears to be mixed, or inconclusive. Both the actual perceptions and expectations play a factor in determining consumer satisfaction or dissatisfaction with food safety, and explain the continued intention among food purchases. In line with this conception, among these food safety incidents in Taiwan, adulterations involving illegal food additives were the most frequent [18]. Items E2 to E7 explain the observed expectation changes in terms of food additives [38].

Food safety and any possible risks related to it, should be known by the consumers, so they can take this information into account in their purchase decisions. Consumers may be aware of the “attention given to the risks of consuming certain foods in Taiwan” (E1) [62], “responsible for food safety after purchase” (E8) [63], or “know where the food originates from” (E9) [40].

In line with these conceptions, consumers may not just blindly trust the system, item E10 “As a result of the occurrence of food safety incidents, I am suspicious about certain food products” is measured for the over-confidence of consumers concerning food safety and public health issues [36]. Each of the above these items are included in Table 4.

## 4. Procedure and Validation of the Food Safety Gap Scale

### 4.1. Sampling and Data Collection

Drawing upon the aforementioned literature regarding food safety, previous research has centralized on improving food safety quality to promote the consumer trust. However, this did not significantly increase consumer confidence in buying foods, mainly because many foods on the market still do not satisfy the consumers’ expectations. Three experts interviewed during this elicitation stage consisted of a member from food manufacturer administration, a senior staff of the government’s health department, and a professor in the field of food safety. Next, a pretest for 30 food consumers to evaluate the comprehension of this closed questionnaire. The participants were recruited from a range of food markets for supermarket, organic stores, and traditional Taiwanese markets. After conducting this pretest, we revised the tested FSGS into the first version for this study.

The first version of the FSGS was distributed during a preliminary and test stage which lasted from December 2017 to January 2018. A total of 215 questionnaires were returned, and only 200 were used for data analysis to evaluate the reliability and validity of each dimension, and we apply confirmatory factor analysis (CFA) to purify the structure of FSGS. The number of indicators were confirmed using the factor loading values greater than 0.7 criterion. Items that did not meet this criterion were removed one at a time to ensure utmost accuracy. After iterative rounds of CFA, we excluded 15 items to constitute the final version.

The final version of the FSG self-assessment questionnaire is divided into four parts, including subjective perceptions of food safety, the importance and performance of food safety, along with basic demographic information of the respondent. All items were scored by using 7-point Likert scale, ranging from 1 = “strong disagree” to 7 = “strong agree”, except for personal information. The data collected were over a period of approximately three months (February 2018–May 2018). A total of 697 valid responses were collected and then included in reliability and validity, and importance–performance analysis (IPA) to understand the food safety gaps of consumers. The profiles of participants were shown in Table 5.

### 4.2. Reliability and Validity

Using the data of subjective perception for reliability and validity analysis. Among the participants in the final version of FSGS (N = 697), the convergent validity was assessed by determining the significance of item to factor loadings [64], and further the internal consistency. The calculated Cronbach’s alpha values ranged from 0.86 to 0.94 (cutoff value >0.70), and displayed in Table 6. Convergent validity was measured by composite reliability (CR), which should be greater than average variance extracted (AVE), which in turn should be greater than 0.5 [65]. The CR values of this study range from 0.90 to 0.95, and AVE value range from 0.59 to 0.76. Thus, good internal consistency among the attributes within each dimension was confirmed. In addition, these thresholds also tested the discriminate validity of the assumption that the AVE square root of each variable exceeds the related coefficients of these variables [65]. Additionally, a pairwise correlation less than 0.85 indicates discriminant validity between two factors [66]. In summary, the 25-item measurement FSG exhibited a considerable construct which was deemed valid and reliable (see Table 6 and Table 7).

## 5. Results of FSG and IPA

Importance–performance analysis (IPA) developed by Martilla and James [67], and marketing research techniques helped provide insights to management in order to identify the strengths and weaknesses of service attributes [68]. More importantly, IPA is able to determine the most important attributes that have the highest impact on consumer satisfaction, and understanding that the lowest performance attributes that need to be improved immediately [69].

Therefore, to date, the application of IPA extends to a wide range of fields, including manufacturing [70], tourism [71,72], hospitality [73], food [74], health care [75], transportation [76] and other industries.

In this study, IPA involves two dimensions and four quadrants that measure the performance/perceived (*x*-axis) and importance/expectation (*y*-axis) of food safety attributes. According to their position on the matrix, the following improvement gap of food safety can be recommended. Quadrant I: “keep up the good work”, are viewed as the opportunities to achieve or maintain competitiveness; Quadrant II: “concentrate here”, high importance, but low performance is considered as a major weakness for food safety gaps. Therefore, immediate improvement is required; Quadrant III: “low priority”, the attributes in this quadrant are not considered crucial and do not require additional efforts; Quadrant IV: “possible overkill”, low importance but high performance, customers do not particularly value these attributes in this quadrant, which indicated that the resources committed are excessive.

### 5.1. Gap Scores and Items Position

Table 8 shows the mean importance (I), mean performance (P), gaps score, paired t-value between importance and performance, and quadrants of each item gained through the analysis of data. The paired sample t-test shows that significant differences exist between the respective expectation mean and perception mean for all 25 items. Moreover, these gap scores between the importance/expectation and performance/actual perceptions are negative which indicates that the quality of food safety is generally less than what consumers think it actually is.

**Figure 2:**
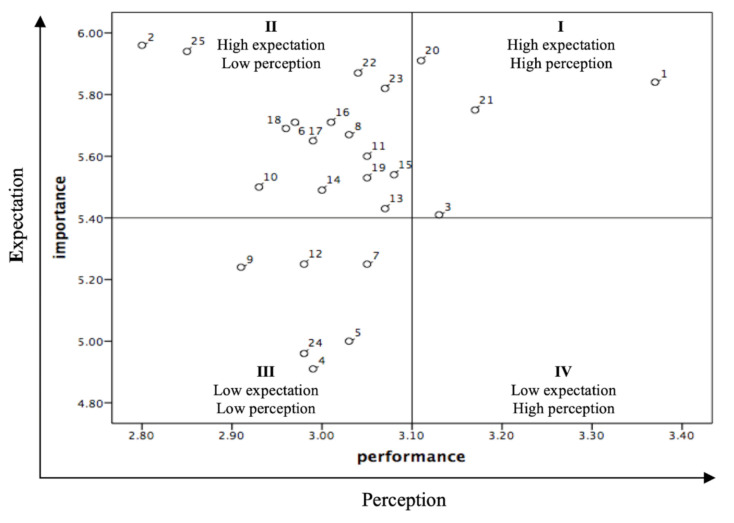
The expectation–perception matrix of food.

From a gap analysis perspective, all items must be improved, as well as the order of their improvement priorities based on the results of the IPA. Looking at the different aspects among score gaps, the items A6 (1.91), A7 (1.96), and E9 (1.97) have a smaller gap score, while items A4 (3.15) and E10 (3.09) have a larger gap score. Specifically, these items mostly belong to the “assurance” and “assessment” constructs in the FSF-scale. As revealed in the above results, the data were then transferred to the IPA matrix presentation in Figure 2. The practical implication based on the result diagram of expectation and perception is that food safety should pay more attention to these items to decrease the gap.

Our findings have revealed that gaps in food safety have highest importance and performance in quadrant I, such as items A3, A5, E1, and E2. Therefore, at least the performance level should be continued and then narrow the gap. In quadrant II, where ‘performance/actual’ perception is lower than ‘importance/expectation’, there are many items that fall into the “concentrate here” quadrant (A4, A9, C3, C5, C9, C12, R1, R2, R5, R6, R7, R8, E7, E8, E10).

In short, government and manufacturers must focus on improving these attributes. It is worth noting that all items related to the “regulation” construct fall within this quadrant. The gap analysis results illustrate that these items with a gap less than 2.33 fall into the “low priority” quadrant (III) in the IPA matrix, implying low importance and low performance. Therefore, these items may not be prioritized when government or manufacturers make improvement plans. In quadrant IV, the consumers are satisfied with the performance but they assess less important food safety attributes. However, not any particular item falls into this quadrant.

### 5.2. The Expectation and Actual Perception Gap

The above results indicate that the gap scores were able to successfully evaluate the magnitude of the gap between consumers’ expectations and actual perceptions of food safety. The evaluation of all items based on expectations and actual perceptions of consumers’ food safety has generated a conflicted consequent with a lower perception and rather higher expectation. This overwhelmingly verifies that a gap exists between consumers’ expectations and actual perceptions of food safety in all indicators, and that the improvement of these indicators needs to be promoted because they fail to satisfy customers’ expectations [77].

**Figure 3:**
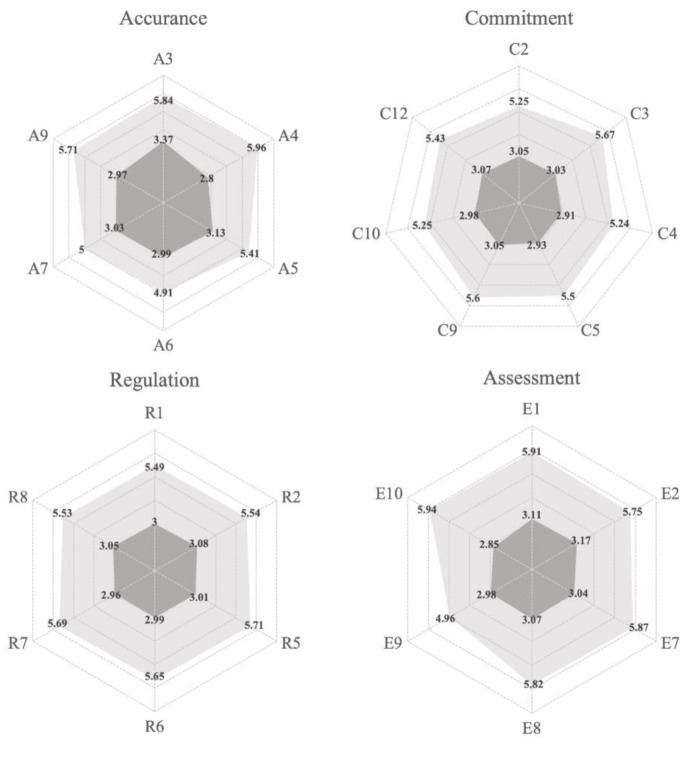
Food safety gaps of all constructs.

Previous studies have argued the gap exists between food safety attitudes and behaviors of respective food vendors [60,61,78]. Our results indicate a gap between consumer expectations and actual perception of food safety, which is one of the enduring challenges for dealing with food safety. Figure 3 represent the magnitude of the gaps within each indicator ranging within the four constructs. From the magnitude in each gap, we found that there is a wide gap existing between expectations and actual perceptions of food safety. Despite most food vendors complying with governmental regulations and standards, it is likely that they cannot truly satisfy the expectations of the consumer.

Moreover, in order to increase the sales within the food economy, the government has inadvertently ignored the actual perception of food safety by consumers and has not yet provided any approach to address consumers’ perceptions of food safety after recurring incidents of food safety. Though recognizing that incident of food safety was an extremely important issue for the future at the consumer level, if manufacturers and governments were unwilling to expand any additional effort in the environment of food, these problems threaten to permanently diminish their quality of food safety.

## 6. Discussion

While food production is becoming increasingly more complex, no matter how different in terms of risk characteristics and the degree of threat to public health, successive food scares may lower consumer confidence. As a result, consumers tend to rely heavily on government and manufacturers to ensure the quality of the products they consume. However, less research has focused on the level of importance consumers place upon aspects of expectation and gap concerns regarding food safety perceptions.

The ECA model is viewed as a three-stage model where first-stage “evaluation” is caused by the gaps between expectations and actual perceptions of food safety, and to confirm the size of the gap. Given this background, the purpose of this paper is to describe the application of a gap model and how it affects and relates to food safety. More specifically, this study was undertaken in order to understand consumers’ gap of food safety, and to suggest some theoretical as well as practical implications of this process.

The results show that Taiwanese consumers have large gaps in the food safety environment. A larger gap between expectations and perceptions means that consumers are less confident about food safety in the market, or their lack of confidence in mandatory regulation has led to the current level of food safety that may not meet the need or expectation of consumers. For instance, IPA suggests the “regulation” performs poorly with all indicators that fall into quadrant II. Therefore, we argue that the government should still be a key actor.

Although the Taiwanese Food and Drug Administration (TFDA) ensures food safety and the quality of food, developed official regulations and analytical methods to assess food quality are used to determine whether food has been adulterated. Even in situations where TFDA affects practice, these influences appear not to be more valid over time. That is, the weak enforcement of food safety regulations, which at best create a favorable attitude. In addition, the consumers from our survey are generally concerned about food safety, they are considered with one of the most important sources, ‘the food labelled with full product information’, since this indicator (A4) has the highest importance score (5.96) and the largest gap score (3.15). The possible reason is that in the initial period of purchasing food, this information can only be obtained through labels seen on the packaging. Therefore, we argue that manufacturers should clearly label food information to reduce potential issues of transparency between consumers and manufacturers.

Even though Taiwan is presently improving its food safety standards, consumer concerns about food safety are also increasing. Indeed, there is a public demand for improving food quality by food providers, the food safety environment is a much more significant factor affecting their decision-making process when they decide to purchase food. In conclusion, food safety has become a priority for many countries, citizens, and the food industry. As food manufacturers place more concern on the strategic importance of food safety, consumers will have a positive view of the governmental regulations, and this is important for consumers. Additionally, consumers are seeking added reassurance and are willing to pay a higher price for a safer food environment.

## 7. Conclusions

The findings of this research should lead to a food safety gap taxonomy which will allow us to study the psychological mechanisms involved when a food safety message is expressed in a consumer environment, versus what is actually communicated from the manufacturer themselves. They could also be applied to improve the importance–performance analysis in food safety, as well as consumer behavior. In general, they will be useful for improving evaluative indicators and in particular, to unify evaluation criteria among the consumer of the food and safety hazards. Furthermore, since food manufacturers are ubiquitous figures in the food market worldwide and are one of the main causes of food safety incidents outbreak, identifying the potential gaps in consumers’ expectations and perceptions is imperative to building upon interventional strategies to promote food safety.

The FSG scale allows quantification of this tendency by evaluating the extent of consumers’ expectations themselves by asserting how they are actually perceived. This research is significant as a new validated scale can now be applied to the measurement of food safety gaps. However, whether this will also apply to food safety contexts in other parts of the world cannot be determined based on this study. Further research is therefore warranted in different cultural and geographical contexts. Finally, this study has some potential limitations. Due to its convenience sampling in Taiwan, the data presented here may not be representative of other regions worldwide. In addition, since this is a cross-sectional survey, we recommend a longitudinal study to obtain more contextual relationships between food safety incidents. Despite these limitations, this study has considerable potential for food safety.

## Figures and Tables

**Table 1:** Indicators of food safety gap between consumers and manufacturers.

Assurance		Source
A1	Food manufacturers have the competence to control the safety of food.	De Jonge et al. [36]
A2	Food manufacturers are honest about the safety of food.	
A3	Food manufacturers are sufficiently open about the safety of food.	
A4	The food product is labelled with all necessary information.	Taylor et al. [40]
A5	The food producer maintains control over the hygiene.	
A6	The shop or retailer maintains control over the hygiene.	
A7	I trust Taiwanese food manufacturers to produce safe foods.	Ariyawardana et al. [21]
A8	I trust processors to honestly convey the country of origin and the product’s ingredients.	
A9	Absence of a code of ethics in enterprises makes you suspect the safety of the food with additives.	Wu et al. [38]
A10	My acquaintances (e.g., family, friends, or colleagues) are alert and attentive to potential problems and risks related to hygiene and food safety.	Griffith et al. [39]

**Table 2:** Indicators of food safety gap between consumers and government.

Commitment		Source
C1	Government’s regulation on additives is lack of effectiveness.	Wu et al. [38]
C2	The government sets clear objectives concerning food safety and hygiene.	De Boeck et al. [25]
C3	The government strives for continuous improvement of food safety and hygiene.	
C4	The government clearly considers food safety and hygiene to be of great importance.	Hall et al. [46]
C5	The government acts quickly to correct problems/issues that affect food safety and hygiene.	
C6	The government sets a good example concerning food safety and hygiene.	Griffith et al. [39]
C7	In our government, the risks related to food safety and hygiene are known.	
C8	In our government, the risks related to food safety and hygiene are under control.	
C9	In our government, good policies and procedures concerning food safety and hygiene are in place.	Yiannas [50]
C10	I think that the food safety level of market product is very poor/very good.	Berry et al. [51]
C11	Taiwan authorities always practice the food safety behaviors they preaches.	Leroy et al. [52]
C12	I think, when the government makes food safety regulations/laws, makes sure manufacturer follow it.	

**Table 3:** Indicators of food safety gap between government and manufacturers.

Regulation		Source
R1	Taiwanese authorities enforce strict hygienic standards for food.	Taylor et al. [40]
R2	I trust imported foods are safe and meet proper standards.	Ariyawardana et al. [21]
R3	I trust the food inspection schemes adopted by the Taiwan government.	
R4	I trust the food safety standards adopted in Taiwan.	
R5	The government communicates regularly with the public about hygiene and food safety.	Yiannas [50]
R6	The government communicates in a clear way with the food manufacturers about food safety and hygiene.	
R7	If health violations have a direct impact on public health and must be corrected immediately. Serious items may, as a group, lead to the closure of a food establishment if not corrected (e.g., food additive, filthy food contact surfaces, temperature problems, food from unapproved sources, adulterated food).	Guchait et al. [37]
R8	These are violations of such a direct and substantial impact to public health that the violation must be immediately corrected or the health officer will require the closure of the food establishment (i.e., no water; no hot water; no ability to sanitize; sewage back up; power outage; pest infestation; no/expired food dealer’s permit).	

**Table 4:** Indicators of food safety gap between consumers’ expectation and actual perceived.

Assessment		Source
E1	I usually put attention on risks of food in Taiwan.	Sparks and Shepherd [62]
E2	Influence from information regarding the presence or absence of quality and safety certification of food with additives.	Wu et al. [38]
E3	Doubt about the food safety of domestic food market.	
E4	Influence from information regarding additives on the package labels.	
E5	Unwilling to buy food with additives due to the experience of family.	
E6	Abuse of food additive became a major potential food risk.	
E7	Lose confidence in the domestic food market.	
E8	Consumer is responsible for food safety after purchase.	Al-Shabib et al. [63]
E9	You know where the food originates from.	Taylor et al. [40]
E10	As a result of the occurrence of food safety incidents, I am suspicious about certain food products.	De Jonge et al. [36]

**Table 5:** Participant characteristics for FSG-scale.

Variables	Items	First Version	Final Version
		CFA (*n* = 200)	IPA (*n* = 697)
Gender	Male	89	376
	Female	111	321
Age	20–30	53	183
	31–40	125	335
	Over 40	22	179
Education	Junior college	28	28
	College	152	442
	Graduate school	20	227
Occupation	Student	21	86
	Government staff	39	89
	Service industry	82	315
	Business	43	131
	Others	15	76

*Note.* CFA = confirmatory factor analysis, IPA = importance–performance analysis.

**Table 6:** FSG items and measurement values.

Constructs	Items	Mean (SD)	Factor Loadings	Cronbach’s α
**Assurance**	A3	5.75 (1.15)	0.816	0.86
	A4	5.88 (0.93)	0.768	
	A5	5.35 (0.95)	0.755	
	A6	4.57 (1.17)	0.754	
	A7	4.75 (1.45)	0.726	
	A9	5.60 (1.21)	0.817	
**Commitment**	C2	5.07 (1.28)	0.836	0.90
	C3	5.60 (1.09)	0.799	
	C4	5.05 (1.29)	0.824	
	C5	5.30 (1.30)	0.731	
	C9	5.52 (1.18)	0.742	
	C10	5.07 (1.27)	0.827	
	C12	5.24 (1.28)	0.765	
**Regulation**	R1	5.34 (1.24)	0.870	0.92
	R2	5.46 (1.07)	0.869	
	R5	5.60 (1.14)	0.874	
	R6	5.52 (1.13)	0.739	
	R7	5.58 (1.14)	0.891	
	R8	5.32 (1.32)	0.861	
**Assessment**	E1	5.86 (1.04)	0.732	0.94
	E2	5.64 (1.18)	0.895	
	E7	5.77 (1.16)	0.951	
	E8	5.75 (1.09)	0.820	
	E9	5.74 (1.22)	0.904	
	E10	5.88 (1.04)	0.923	

**Table 7:** Results of correlation, reliability, and validity.

Constructs	*M*	*SD*	1	2	3	4	CR	AVE
1. Assurance	5.32	0.85	**0.77**				0.90	0.59
2. Commitment	5.26	0.98	0.66 **	**0.79**			0.92	0.62
3. Regulation	5.47	1.00	0.71 **	0.76 **	**0.85**		0.94	0.72
4. Assessment	5.77	0.98	0.70 **	0.70 **	0.82 **	**0.87**	0.95	0.76

*Note.* Items on the diagonal in boldface represent the AVE square root; ** *p* < 0.01.

**Table 8:** Result of IPA.

IPA Code	Item Information	I	P	Gaps (P-I)	Paired *t*-Value	Quad.
**Assurance**						
1	A3. Sufficiently open about the safety of food.	5.84	3.37	−2.46	49.93 ***	(I)
2	A4. Labelled with all necessary information.	5.96	2.80	−3.15	61.70 ***	(II)
3	A5. The food producer maintains control over the hygiene.	5.41	3.13	−2.28	46.25 ***	(I)
4	A6. The shop or retailer maintains control over the hygiene.	4.91	2.99	−1.91	38.75 ***	(III)
5	A7. I trust Taiwanese food manufacturers.	5.00	3.03	−1.96	37.95 ***	(III)
6	A9. Absence of a code of ethics in enterprises.	5.71	2.97	−2.73	50.83 ***	(II)
**Commitment**						
7	C2. The government set clear objectives.	5.25	3.05	−2.19	39.30 ***	(III)
8	C3. The government strives for continuous improvement.	5.67	3.03	−2.64	50.70 ***	(II)
9	C4. Food safety and hygiene to be of great importance.	5.24	2.91	−2.32	43.01 ***	(III)
10	C5. The government acts quickly to correct problems.	5.50	2.93	−2.56	46.44 ***	(II)
11	C9. In our government, policies, and procedures are in place.	5.60	3.05	−2.54	49.06 ***	(II)
12	C10. Food safety level.	5.25	2.98	−2.26	42.21 ***	(III)
13	C12.The manufacturer follow regulations/laws.	5.43	3.07	−2.36	43.80 ***	(II)
**Regulation**						
14	R1. Strict hygienic standards for food.	5.49	3.00	−2.48	47.33 ***	(II)
15	R2. Imported food are safe.	5.54	3.08	−2.46	47.57 ***	(II)
16	R5. Communicate regularly with the public.	5.71	3.01	−2.69	52.21 ***	(II)
17	R6. Communicate clearly with the food manufacturers.	5.65	2.99	−2.65	51.24 ***	(II)
18	R7. Health violations and must be corrected immediately.	5.69	2.96	−2.72	54.67 ***	(II)
19	R8. If violations will require the closure.	5.53	3.05	−2.48	49.26 ***	(II)
**Assessment**						
20	E1. Attention on risks of food in Taiwan.	5.91	3.11	−2.79	55.56 ***	(I)
21	E2. Quality and safety certification of food with additives.	5.75	3.17	−2.58	53.52 ***	(I)
22	E7. Confidence in the domestic food market.	5.87	3.04	−2.82	53.97 ***	(II)
23	E8. Responsible for food safety after purchase.	5.82	3.07	−2.75	54.33 ***	(II)
24	E9. Know where the food originates from.	4.96	2.98	−1.97	44.10 ***	(III)
25	E10. Food safety incident caused doubt of food.	5.94	2.85	−3.09	62.24 ***	(II)

*Note.* I = Importance, P = performance, Quad. = Quadrant, no item in quadrant IV, *** *p* < 0.001 significance levels.
